# Radiotherapy for the Palliation of Advanced Sarcomas—The Effectiveness of Radiotherapy in Providing Symptomatic Improvement for Advanced Sarcomas in a Single Centre Cohort

**DOI:** 10.3390/healthcare7040120

**Published:** 2019-10-18

**Authors:** Hannah Tween, David Peake, David Spooner, Jenny Sherriff

**Affiliations:** Hall-Edwards Radiotherapy Research Group, Queen Elizabeth Hospital, Birmingham, B15 2WB, UK; David.Peake@uhb.nhs.uk (D.P.); David.Spooner@uhb.nhs.uk (D.S.); Jenny.Sherriff@uhb.nhs.uk (J.S.)

**Keywords:** sarcoma, radiotherapy, palliative

## Abstract

*Background:* Sarcomas are rare and heterogeneous tumours with a large proportion of patients requiring palliative intervention. They are regarded as relatively radioresistant and therefore achieving good palliation with radiation may require larger doses than for more common solid tumour types. Limited data is available regarding appropriate palliative radiotherapy dose fractionation. This case series aims to assess the effectiveness of radiotherapy in providing symptomatic improvement for advanced sarcomas. *Method*: Data was retrospectively collected for patients treated with palliative radiotherapy between July 2010 and April 2019 at one institution. The primary outcome was documented symptomatic improvement following radiotherapy. Secondary outcome was overall survival. *Results*: One hundred and five patients had a total of 137 sites treated using 25 different dose fractionation schedules. The median patient age was 54 (range 8–90) years. Treated sites included 114 soft tissue and 23 bone sarcomas. Data on symptomatic improvement was available in 56% and 67% of cases respectively. A total of 70% of soft tissue and 55% of bone sarcoma patients reported symptomatic improvement. Symptomatic response rates appeared to increase to a biological effective dose (BED) of 50Grey_4_ (Gy_4_) (alpha beta ratio (α/β) = 4 for tumour) but did not continue to improve with further rises in dose beyond this. *Conclusion*: Palliative radiotherapy offers symptomatic improvement for sarcoma patients with two-thirds of patients reporting reduction in symptoms. These results are limited by the heterogeneous study population including different sarcoma subtypes each with a probable different radio-sensitivity, treated with different radiotherapy schedules. Further prospective data collection is needed considering sarcoma subtype radio-sensitivity, to determine appropriate palliative dose fractionation schedules.

## 1. Introduction

Bone and soft tissue sarcomas are rare, accounting for approximately 1% of all malignancies. In the United Kingdom, 3298 new soft tissue sarcomas and 531 bone sarcomas were diagnosed in 2010 [1]. They comprise a heterogeneous group of tumours, with over 100 different histological subtypes [2]. The diagnosis can often be difficult and delayed; hence a large proportion of patients will require palliative intervention during the course of their disease. Approximately 50% of patients with high grade soft tissue sarcoma will develop metastatic disease requiring palliative treatment [3,4,5], most commonly with systemic chemotherapy. In addition, radiotherapy may have a role in the palliation of symptoms. A review of sarcoma services in Australia demonstrated that 36% of patients with metastatic disease were offered palliative radiotherapy [6].

Sarcomas are commonly regarded as being relatively radio-resistant, with doses of 60–70 Gy in 2 Gy per fraction required in the radical setting to control microscopic disease. Therefore, it could be hypothesized that achieving good palliation with radiation may require larger doses than are used for more common solid tumour types. To date, limited data is available in the published literature regarding appropriate palliative radiotherapy dose fractionation schedules for sarcoma. Furthermore, there is evidence that different histological subtypes of sarcoma have different response rates to commonly prescribed chemotherapeutic agents [7,8] and therefore it seems likely that the same is true for these tumours inherent radio-sensitivity. Some histological subtypes may potentially require larger doses to achieve good palliation compared to others. The rarity and heterogeneity of sarcomas makes this very difficult to research and therefore limited data is currently available to guide the appropriate palliative radiotherapy dose fractionation schedules for the different subtypes of bone and soft tissue sarcoma. In addition, these tumours can present as primary or secondary tumours at any anatomical site, adding further complexities when prescribing palliative radiotherapy as the organs at risk and their normal tissue tolerances can vary greatly.

Symptoms requiring palliation in patients with metastatic sarcoma are no different to those suffered by patients with other metastatic solid tumour types and include pain (both from bony and soft tissue tumours), neurological symptoms from brain or spinal metastases, bleeding or superior vena cava obstruction. A previously published case series considered 17 patients with symptomatic metastatic sarcoma requiring rapid palliation [9]. They were treated with a hypofractionated regime with 39 Gy in 13 fractions, treating daily Monday to Friday and reported that this was well tolerated. At a median follow-up of approximately 6 months, the majority achieved durable pain control. Similarly, a review of four patients treated with palliative radiotherapy for metastatic spinal cord compression secondary to soft tissue sarcoma revealed a successful rate of pain control with standard palliative doses of radiation (20 Gy in 5 fractions or 30 Gy in 10 fractions), however the effect on preservation of motor function was less successful and the authors concluded that upfront neurosurgery is required to have any benefit on neurological function [10].

The aim of this case series was to assess the effectiveness of different radiotherapy dose and fractionation regimens in providing symptomatic improvement in patients with advanced and metastatic sarcomas at one institution.

## 2. Materials and Methods

Patients receiving radiotherapy for sarcoma with palliative intent, at University Hospitals Birmingham Birmingham, United Kingdom between July 2010 and April 2019 were retrospectively identified from a prospective database. Patients were included if they had a histologically confirmed soft tissue or bone sarcoma and had provided informed consent for palliative radiotherapy.

Data collected included demographics, bone or soft tissue sarcoma status, histological subtype of sarcoma, indication for radiotherapy, site treated and details of the dose fractionation schedule of radiation offered and delivered. Dose fractionation schedules were selected by the treating clinician based upon the histological subtype of sarcoma, with the aim of giving a higher biologically effective dose for less radiosensitive subtypes. Furthermore, the indication for radiotherapy also influenced the chosen dose fractionation schedule, with a higher biological effective dose (BED) often used for patients with a better prognosis. Radiotherapy was planned with either simulation, virtual simulation or a conformal planning technique. Radiotherapy was delivered using a single field, parallel opposed fields, a conformal plan, an intensity modulated radiotherapy plan or a stereotactic ablative body radiotherapy (SABR) plan. The majority of patients were treated with 6-MV or 10-MV photons delivered using a linear accelerator; however, a small number of patients were treated with SABR (CyberKnife).

### 2.1. Outcomes

The primary outcome was documented symptomatic improvement following radiotherapy. Clinical and electronic patient records were retrospectively reviewed considering the baseline symptoms and the main indication for radiotherapy, as well as documented improvement in these symptoms following radiotherapy. Symptomatic improvement was defined by clear documentation that the symptom had either resolved or improved in the three months after radiotherapy. Given the retrospective nature of this study no attempt was made to further quantify the degree of improvement of the symptoms as this could not be accurately measured. Secondary outcome was overall survival.

### 2.2. Statistics

Overall survival was calculated from the time of completion of radiotherapy to the time of death or last follow-up. An alpha beta ratio of 4 was used for the tumour, as per previous publications [9] to calculate the biological effective dose of each dose fractionation schedule used, to allow a comparison of these schedules. The study was registered with the hospital clinical governance committee (reference number: CARMS-15316).

## 3. Results

A total of 105 patients were identified as having received palliative radiotherapy between July 2010 and April 2019 with a total of 137 sites treated. Baseline tumour characteristics are shown in Table 1. Of the sites treated 114 sites were soft tissue sarcomas and 23 were bone sarcomas. The median age at the time radiotherapy was deliveredwas 54 (range 8–90) years.

**Figure 1:**
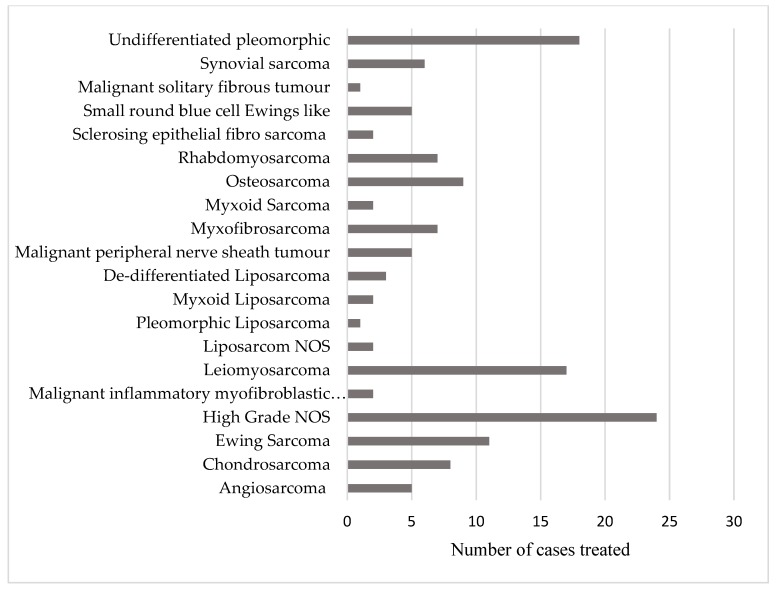
This figure demonstrates the number of cases treated for each histological subtype.

**Figure 2:**
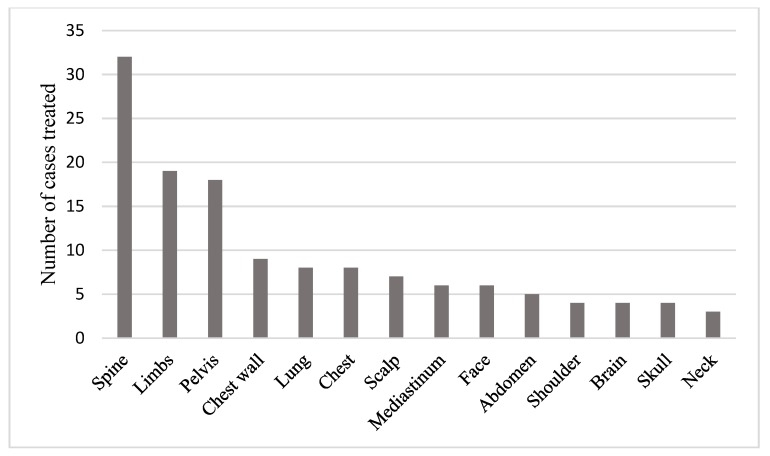
This figure demonstrates the number of cases treated in each anatomical site.

The patient population included 17 subtypes of soft tissue sarcoma and 3 subtypes of bone sarcoma (Figure 1). A total of 15 different anatomical sites were treated with palliative radiotherapy during the time period studied. These are shown in Figure 2. Table 2 and Table 3 shows the details of the radiotherapy dose fractionation schedules delivered, and the radiotherapy techniques used. A total of 25 different dose fractionation schedules were used.

The commonest indication for treatment was pain (*n* = 74). Other indications included breathlessness (*n* = 5), fungating tumour (*n* = 7), metastatic spinal cord compression (*n* = 6), bleeding (*n* = 4), neurological dysfunction (*n* = 3), symptomatic brain metastases (*n* = 2), cough (*n* = 1), and superior vena cava obstruction (*n* = 1). Several patients were given a higher dose of palliative radiotherapy for local control (*n* = 23).

Data on symptomatic improvement was available in 56% of the soft tissue sarcomas and 67% of the bone sarcomas treated. A total of 70% of soft tissue and 55% of bone sarcoma patients reported symptomatic improvement. Twenty Gy in 5 fractions was the most commonly used schedule with 50% of patients with soft tissue sarcomas reporting an improvement in symptoms, whilst 67% of patients with bone sarcoma reported an improvement.

**Figure 3:**
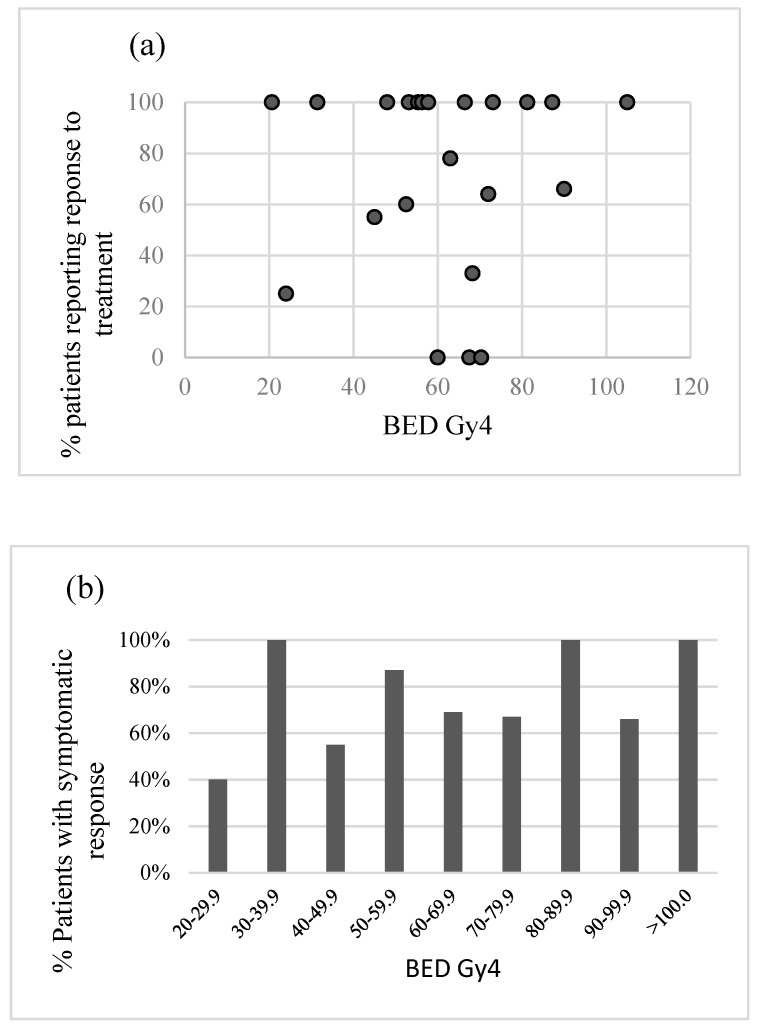
This figure demonstrates symptomatic response based on BED. (**a**) Demonstrates the response for each separate dose and fractionation regimen, expressed as its BED (Gy_4_); (**b**) demonstrates the response for all dose and fractionation regimens with a BED within each given range.

Because of the large variation in dose and fractionations used, each schedule was converted to biological effective dose (BED) to allow comparison between schedules. An alpha beta ratio (α/β) of 4 for the tumour was used as this has been used in a previous case series [9]. Table 4 illustrates the BED calculation and Figure 3 demonstrates the symptomatic response compared to the BED.

Because of the small numbers of patients in each histological subtype it is difficult to analyse the response to radiotherapy based on subtype. However, response rates were higher for soft tissue sarcomas with all patients with angiosarcoma and leiomyosarcoma reporting an improvement in symptoms. Response was lowest in osteosarcomas and chondrosarcomas with only 25% of patients reporting an improvement in symptoms.

**Figure 4:**
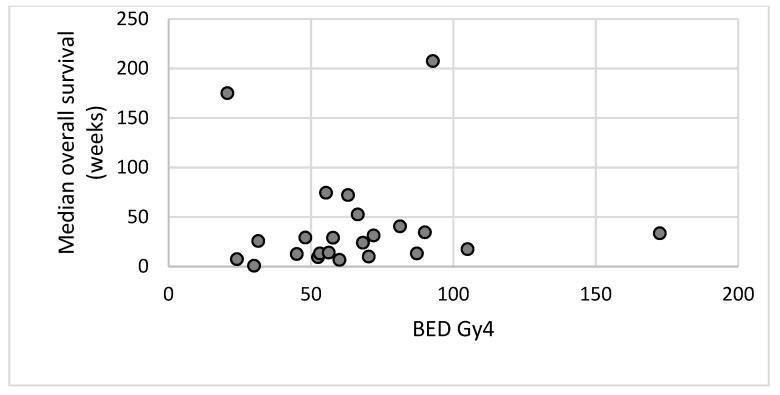
This figure demonstrates the median overall survival of patients plotted against BED for each dose/fractionation regime.

Survival data was available for 129 sites treated. This was calculated from the date of completion of radiotherapy to the date of death or last follow-up. Figure 4 illustrates median overall survival versus the biological effective dose of the radiotherapy delivered.

## 4. Discussion

There is little published literature considering the role of palliative radiotherapy in metastatic sarcoma, although it is recommended as a treatment option for palliation by both the UK bone and soft tissue sarcoma guidelines [7,8] and the European Society of Medical Oncology (ESMO) guidelines [11,12]. To the authors’ knowledge this is the largest retrospective study published to date reviewing the use of palliative radiotherapy for sarcoma. The results confirm that palliative radiotherapy can successfully provide symptomatic benefit to patients with metastatic sarcoma. This study is limited by being retrospective such that the assessment of symptomatic improvement could only be assessed from documentation in medical records. It was therefore not possible to quantify the amount of symptomatic improvement seen or indeed to be certain of the accuracy of the results. The aim of this review, however, was to confirm that radiotherapy does have a role in the palliation of advanced sarcomas and to guide future prospective studies to gain more accurate evidence of the use of palliative radiotherapy in this setting. In addition, this study was limited by the heterogeneous population studied. Both soft tissue and bone sarcomas of multiple types were included. The radiobiology of the different subtypes of sarcoma has not been well studied, but it is accepted that these tumours tend to be relatively radio-resistant, with a high alpha beta ratio. There have been several publications considering the role of palliative radiotherapy for other, more common solid tumour types. A meta-analysis has shown that when treating bone metastases from a number of different primary tumour sites and histology’s, there appears to be little short-term symptomatic benefit in a fractionated course of radiotherapy, compared to a single fraction [13]. Most oncologists therefore advocate the use of a single fraction to control pain secondary to bone metastases [14]. With longer follow-up, however, it seems that patients treated with a fractionated course of palliative radiotherapy are less likely to need retreatment than those given a single fraction [13]. This study demonstrates that selected patients with advanced sarcoma can benefit from palliative radiotherapy provided their prognosis is long enough for the benefits from radiotherapy, in terms of symptomatic improvement, to outweigh the inconvenience of undergoing radiotherapy and the associated side effects that they may suffer.

As sarcomas are felt to be intrinsically radio-resistant, it can be hypothesized that a single fraction of palliative radiotherapy may not be sufficient to offer adequate symptomatic benefit. The results presented here suggest a higher symptomatic response rate with a biological effective dose (BED) of 50 or greater. Figure 3b demonstrates an increase in response rate with increasing BED up to 50 Gy_4_. Beyond this point the response is maintained but does not appear to increase further, suggesting very high doses of palliative radiotherapy may not be necessary to achieve a good symptomatic response in this patient group. The one apparent outlier in Figure 3b is the 100% symptomatic response in patients receiving a BED of 30–39.9, this is likely to be an outlier as there was only one patient in this group.

Because of the limited evidence available in the use of radiotherapy for palliation in sarcoma the Royal College of Radiologists Radiotherapy dose fractionation guidance (3rd edition) [15] recommends several different dose fractionation schedules (Table 5). Excluding 8 Gy single fraction and 20 Gy in 5 fractions which are commonly used schedules for all solid tumours; the remaining recommended dose fractionation schedules all have a BED of greater than 50 Gy_4_. Although this case series has demonstrated that very high doses of radiotherapy may not provide additional short-term symptomatic benefit, longer courses of radiotherapy may be considered in patients with a good performance status and prognosis in an attempt to provide a longer period of symptomatic benefit without the need for retreatment. A previously published case series of 17 patients who received palliative radiotherapy for sarcoma using 39 Gy in 13 fractions over two and a half weeks demonstrated this was well tolerated and provided high rates of durable pain control [9]. Collection of survival data may help to assess if the correct patients are being offered higher dose, longer courses of radiotherapy. Figure 4 however does not appear to show any correlation between the BED of the radiotherapy delivered and overall survival. Interpretation of this data is extremely limited because of the heterogeneity of the patient population with multiple different histological subtypes and the small numbers of patients treated with each dose and fractionation schedule.

To gain a better understanding of the most appropriate dose and fractionation schedules for different sarcoma subtypes large volume, multicentre, prospective data collections is required using a small number of different dose and fractionation schedules, ideally those outlined by the Royal College of Radiologists (Table 5) [15].

## 5. Conclusions

Palliative radiotherapy offers symptomatic improvement for sarcoma patients. These results are limited by the heterogeneous study population which includes different sarcoma subtypes with different radio-sensitivities, treated with different radiotherapy schedules. Despite this radiotherapy appears to be an effective treatment for symptom control with two-thirds of patients reporting symptomatic improvement. Further multicentre prospective data collection is needed considering the sarcoma subtype radio-sensitivity, to determine appropriate palliative dose fractionation schedules.

## Figures and Tables

**Table 1:** Patient and tumour characteristics.

Patient Characteristic	Patient No.
*Median age(years) at time of radiotherapy*	
(range)	54 (8–90)
*Gender*	
Male	65
Female	40
*Type of Sarcoma*	
Soft tissue	114
Bone	23

**Table 2:** Dose and fractionation schedules used.

Bone Sarcomas		Soft Tissue Sarcoma			
Dose Fractionation	*n*	Dose Fractionation	*n*	Dose Fractionation	*n*
8 Gy 1#	2	8 Gy 1#	9	30 Gy 10#	9
17 Gy 2#	1	12 Gy 2#	1	35 Gy 15#	3
20 Gy 5#	6	15 Gy 10#	1	36 Gy 6#	3
30 Gy 3#	3	16 Gy 2#	2	36 Gy 9#	12
30 Gy 10#	4	17 Gy 2#	1	36 Gy 12#	11
35 Gy 15#	1	17 Gy 5#	1	39 Gy 13#	4
36 Gy 12#	2	20 Gy 5#	29	40 Gy 15#	4
39 GY 13#	1	21 Gy 3#	6	45 Gy 12#	1
50 Gy 20#	1	24 Gy 4#	1	45 Gy 20#	1
50.4 Gy 28#	1	25 Gy 5#	6	50 Gy 20#	3
60 Gy 8#	1	30 Gy 6#	1		

n—no. of patients treated, #—no. of radiotherapy fractions treatment delivered over.

**Table 3:** Radiotherapy technique used.

Technique Used	Number of Patients
Single field	39
Opposed fields	50
Planned volume	36
SABR VMAT	5
SABR Cyberknife	1
Unknown	6

SABR—Stereotactic Ablative Body Radiotherapy, VMAT—Volumetric modulated arc therapy.

**Table 4:** Dose and fractionation regimens expressed as biological effective dose (BEDGy_4_).

Dose and Fractionation	BEDGy_4_	Dose and Fractionation	BEDGy_4_
15 Gy 10#	20.63	36 Gy 12#	63
8 Gy 1#	24	40 Gy 15#	66.43
12 Gy 2#	30	30 Gy 6#	67.5
17 Gy 5#	31.45	39 Gy 13#	68.25
20 Gy 5#	40	45 Gy 20#	70.31
16 Gy 2#	48	36 Gy 9#	72
30 Gy 10#	52.5	50.4 Gy 28#	73.08
17 Gy 2#	53.13	50 Gy 20#	81.25
35 Gy 15#	55.39	45 Gy 12#	87.19
25 Gy 5#	56.25	36 Gy 6#	90
21 Gy 3#	57.75	30 Gy 3#	105
24 Gy 4#	60	60 Gy 8#	172.5

Gy—Radiotherapy dose in Grey #—no. of radiotherapy fractions treatment delivered over.

**Table 5:** Royal College of Radiologist recommended palliative dose and fractionation schedules for sarcoma.

Total Dose (Gy)	Number of Fractions	Length of Treatment	Biological Effective Dose (Gy4)
8	1	1 day	24
20	5	1 week	40
30	5	5 weeks	75
30	10	2 weeks	52.5
36	12	2.5 weeks	63
39	13	2.5 weeks	68.25
40	15	3 weeks	66.70

From Royal College of Radiologists, Radiotherapy Dose fractionation, 3rd edition, March 2019, Chapter 14 Sarcoma [15].
